# Identifying a predictive model of cognitive impairment in bipolar disorder patients: a machine learning study

**DOI:** 10.1192/j.eurpsy.2023.1276

**Published:** 2023-07-19

**Authors:** C. Monopoli, L. Fortaner-Uyà, F. Calesella, F. Colombo, B. Bravi, E. Maggioni, E. Tassi, I. Bollettini, S. Poletti, F. Benedetti, B. Vai

**Affiliations:** 1 IRCCS San Raffaele Hospital, Psychiatry and Clinical Psychobiology Unit; 2 University Vita-Salute San Raffaele; 3 Politecnico di Milano, Department of Electronics - Information and Bioengineering; 4 Department of Neurosciences and Mental Health, Fondazione IRCCS Ca’ Granda Ospedale Maggiore Policlinico, Milan, Italy

## Abstract

**Introduction:**

Bipolar patients (BP) frequently have cognitive deficits, that impact on prognosis and quality of life. Finding biomarkers for this condition is essential to improve patients’ healthcare. Given the association between cognitive dysfunctions and structural brain abnormalities, we used a machine learning approach to identify patients with cognitive deficits.

**Objectives:**

The aim of this study was to assess if structural neuroimaging data could identify patients with cognitive impairments in several domains using a machine learning framework.

**Methods:**

Diffusion tensor imaging and T1-weighted images of 150 BP were acquired and both grey matter voxel-based morphometry (VBM) and tract-based white matter fractional anisotropy (FA) measures were extracted. Support vector machine (SVM) models were trained through a 10-fold nested cross-validation with subsampling. VBM and FA maps were entered separately and in combination as input features to discriminate BP with and without deficits in six cognitive domains, assessed through the Brief Assessment of Cognition in Schizophrenia.

**Results:**

The best classification performance for each cognitive domain is illustrated in Table 1. FA was the most relevant neuroimaging modality for the prediction of verbal memory, verbal fluency, and executive functions deficits, whereas VBM was more predictive for working memory and motor speed domains.

**Table 1.** Performance of best classification models.

	**Input feature**	**Balance Accuracy (%)**	**Specificity (%)**	**Sensitivity (%)**
**Verbal Memory**	FA	60.17	51.31	43
**Verbal Fluency**	FA	57.67	62	53.33
**Executive functions**	FA	60	63.33	56.67
**Working Memory**	VBM	56.50	56	57
**Motor speed**	VBM	53.50	47.67	59.33
**Attention and processing speed**	VBM + FA	58.33	49.17	67.5

**Conclusions:**

Overall, the tested SVM models showed a good predictive performance. Although only partially, our results suggest that different structural neuroimaging data can predict cognitive deficits in BP with accuracy higher than chance level. Unexpectedly, only for the attention and processing speed domain the best model was obtained combining the structural features. Future research may promote data fusion methods to develop better predictive models.

**Disclosure of Interest:**

None Declared

